# Age and Serum Creatinine Can Differentiate Wilson Disease Patients with Pseudonormal Ceruloplasmin

**DOI:** 10.1155/2023/9344891

**Published:** 2023-03-02

**Authors:** Lin Chen, Yongguang Shi, Nan Wang, Zhuoqi Lou, Liya Pan, Xiaolan Xu, Chensi Wu, Yongzhu Han, Renmin Yang, Wenbin Hu, Bing Ruan

**Affiliations:** ^1^State Key Laboratory for Diagnosis and Treatment of Infectious Diseases, National Clinical Research Center for Infectious Diseases, Collaborative Innovation Center for Diagnosis and Treatment of Infectious Diseases, The First Affiliated Hospital, Zhejiang University School of Medicine, Hangzhou, Zhejiang Province, China; ^2^Department of Neurology, The Affiliated Hospital of the Neurology Institute of Anhui University of Chinese Medicine, Hefei, Anhui Province, China; ^3^Department of Geriatric Endocrinology, The First Affiliated Hospital of Anhui Medical University, Hefei, Anhui Province, China

## Abstract

**Methods:**

We retrospectively screened individuals with serum Cp ≥ 140 mg/L from 1032 WD patients who were hospitalised for the first time. Logistic regression analyses were performed in a case-control study between the WD cohort and another liver disease cohort to explore the independent risk factors for WD diagnosis and establish a regression model to identify them. The follow-up medical records of the WD cohort were subjected to mixed-effects model analysis in a longitudinal study to discover factors associated with Cp normalisation.

**Results:**

Eighty-six WD patients and their 353 medical records and another 98 non-WD liver disease patients were included in the present study. Cp normalisation was significantly associated with the copper burden and liver function indexes, such as urinary copper, *γ*-glutamyltransferase, and albumin (*p* ≤ 0.001). Logistic regression analysis showed that age and serum creatinine (*p* ≤ 0.001) were independent risk factors associated with WD. The AUC value of the regression model in the total cohort was 0.926 (*p* ≤ 0.001). At a cutoff value of ≥0.617 and ≥−1, the positive and negative predictive values were both 90.8% for WD.

**Conclusion:**

Increased serum Cp in WD patients is related to excessive copper burden and hepatic injury, and common tests can effectively distinguish WD patients from other liver injury patients.

## 1. Introduction

Wilson disease (WD) is an autosomal recessive copper metabolism disease caused by mutations in the *ATP7B* gene, which encodes a copper-transporting P-type ATPase to convey copper for synthesising ceruloplasmin (Cp) [1, 2]. The decrease in serum Cp concentration is a useful diagnostic hallmark of WD [2]. An epidemiological investigation revealed that the incidence and prevalence of WD in China were approximately 1.96/100,000 and 5.87/100,000, respectively [3]. It is estimated that 50% of patients with active Wilson's liver disease and 15–36% of children with WD may have Cp levels in the low normal range [4–6]. Serum Cp is mainly produced by hepatocytes, and in addition, cells in several other secretory organs, such as the kidney, mammary gland, placenta, and the choroid plexus of the brain, also produce Cp [7, 8]. In addition, macrophages and mononuclear cells in blood during inflammation also have the above function [9, 10]. Therefore, active liver disease, pregnancy, and inflammation will increase Cp, while kidney failure and end-stage liver disease will be just the opposite. The normal concentration of Cp measured by the enzymatic assay has a lower limit of 200 mg/L [6]. The diagnostic accuracy for WD using a cutoff value of 140 mg/L had 100% positive predictive values and 97.1% negative predictive values [11]. In WD, it is usually lower than 100 mg/L, especially in neurologic WD. However, it may be in the low normal range in hepatic WD with active liver disease [12–14] and correlated with liver histologic activity [15]. Because these WD patients' manifestations and serum Cp overlap with those of other liver diseases, their diagnosis may be missed.

Some laboratory findings and indexes are helpful in distinguishing WD with acute liver failure (ALF) from non-WD ALF, including increased aspartate aminotransferase: alanine aminotransferase (AST : ALT) ratios, low serum alkaline phosphatase (AP) activity, and decreased AP to total bilirubin (TB) ratios [13, 16, 17]. However, in the non-ALF WD patients with nearly normal Cp, the indicators that are different from those of other etiologies of hepatopathy have not yet been studied.

Here, we retrospectively screened 1032 WD patients, enrolled 86 individuals who had nearly normal serum Cp, and analysed their subsequent multiple medical records to identify factors related to serum Cp pseudonormalisation. In addition, according to logistic regression analysis, we compared the control group to develop a regression model to differentiate WD from other hepatopathy patients [18].

## 2. Materials and Methods

### 2.1. WD Patient Cohort

We retrospectively investigated 1032 WD patients who were first hospitalised in the affiliated hospital of the Neurology Institute of the Anhui University of Chinese Medicine (Hefei city, Eastern China) from March 2010 to July 2013. The enrolled criteria included (1) meeting the Leipzig criteria for WD diagnosis [16, 19], (2) serum Cp concentration being ≥140 mg/L at diagnosis [11], and (3) patients being younger than 65 years. The following cases were excluded: (1) pregnancy, liver [20] or renal failure [21], chronic kidney disease, infection, cancer, and alcohol abuse; (2) other suspicious hepatitis such as viral or autoimmune hepatitis and drug-induced liver injury; and (3) incomplete medical records.

**Figure 1:**
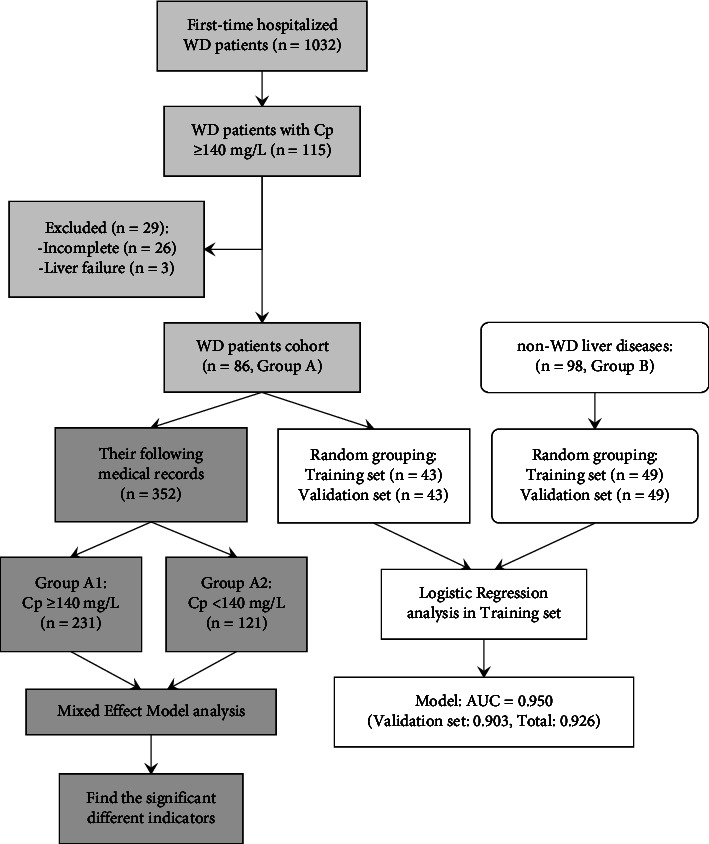
The flow chart of this study.

Finally, 86 WD patients (group A) were enrolled in this study. We followed up all of their electronic medical information as of December 2019 and obtained 352 records. To understand which indicators are associated with changes in serum Cp, in light of the serum Cp concentration, 352 medical records were classified as group A1 (Cp ≥ 140 mg/L, *n* = 231) and group A2 (Cp < 140 mg/L, *n* = 121) to carry out a longitudinal study, regardless of which patient they belonged to. The flow chart is shown in Figure 1.

Their usual information and laboratory results, including sex, age, duration of onset, and follow-up, phenotype, Kayser–Fleischer ring (K–F ring), laboratory biochemical findings, serum Cu and Cp, serum ceruloplasmin oxidase (Sco), 24-hour urinary copper, Child–Pugh scores and classification, abdominal ultrasonographic or radiological findings, liver cirrhosis or not, and mutational sites of the *ATP7B* gene, were recorded. *ATP7B* was detected with next-generation DNA sequencing. AfterQC [22] was applied to generate “clean reads” for further analysis. Then, the “clean reads” (with a length of 150 bp) were aligned to the human genome reference (hg19) using Burrows Wheeler Aligner (BWA) software [23]. After alignment, SNVs and indels were detected using Genome Analysis Toolkit (GATK) software [24]. All SNVs and indels were filtered and estimated via multiple databases, including 1000 Genomes (1000 human genome dataset), gnomAD (Genome Aggregation Database) dataset, and ExAC (Exome Aggregation Consortium) dataset. Serum copper was determined by using flame atomic absorption spectrophotometry [25]. Serum Cp was measured by the immunonephelometric assay with antiserum to human Cp [26]. Sco activity with o-dianisidine dihydrochloride as a substrate was determined by spectrophotometry [26, 27]. The basic 24-hour urinary copper and with intravenous infusion of dimercaptopropane sulfonate (DMPS) sodium or oral penicillamine was recorded.

### 2.2. Case-Control Cohort of Non-WD Liver Disease Patients

WD patients with Cp normalisation would overlap with that of other liver patients, so we included the control cohort of non-WD liver disease patients to conduct logistic regression analysis and establish a model to distinguish WD patients.

The control group patients came from the liver disease department of First Affiliated Hospital, Zhejiang University School of Medicine, in March 2021. Those patients were included if they met the following criteria: (1) identifiable acute liver diseases and compensated or decompensated liver cirrhosis (LC) due to varied non-WD causes, (2) serum Cp concentration ≥140 mg/L, and (3) <65 years. The exclusion criteria included pregnancy, liver or renal failure, chronic kidney disease, infection, and cancer. Finally, 98 liver injury patients were included in this control cohort, including 23 nonalcoholic steatosis hepatitis (NASH), 27 acute hepatitis (hepatitis B: 11, hepatitis E: 7, medicamentous: 5, autoimmune hepatitis: 2, and Epstein–Barr virus infection and hyperthyreosis: 1 for each), 4 compensated LC induced by hepatitis virus B (HBV), and 44 decompensated LC (induced by CHB: 38, alcohol: 2, and CHB + alcohol, medicamentous, primary sclerosing cholangitis, and Budd–Chiari syndrome: 1 for each).

### 2.3. Statistical Analysis

All statistical analyses were performed with SPSS version 21.0 (IBM, New York, USA). The chi-square and nonparametric tests, *t*-test, and ANOVA were carried out, respectively, depending on different data types. A generalized linear mixed-effects model (GLMM) was conducted on repeated measurement data (group A1 vs. A2, Figure 1) to identify indicators related to an increase in Cp. WD and non-WD case-control cohorts (group A vs. B, Figure 1) were divided into training and validation sets for conducting logistic regression analysis to develop a model and validate it. Factors that revealed significant differences in the univariate analysis (*p* < 0.05) were then included in the multiple binary logistic regression analysis with the backward method to identify independent factors associated with WD and develop a regression model. The area under the receiver operating characteristic curve (AUC) was used to evaluate the diagnostic value of the logistic regression model. The sensitivity, specificity, positive predictive value (PPV), and negative predictive value (NPV) of the model were calculated to determine the optimal and handy cutoff values.

## 3. Results

### 3.1. Baseline Characteristics of 86 WD Patients

The baseline characteristics and laboratory findings of 86 enrolled WD patients (male: 42, 48.8%) at the first admission are shown in Table 1. There were three phenotypes: hepatic type (H-type, *n* = 51, 59.3%), hepatic and neurologic presentations (HN-type, *n* = 27, 31.4%), and neurologic type (N-type, *n* = 8, 9.3%). None of the patients converted to other types during the follow-up period. Comparing the three phenotypes, there were no differences in age at presentation (*p*=0.091), but the age at diagnosis especially varied between them (*p*=0.005). Therefore, the N-type had the longest undiagnosed duration and duration of onset, 12.5 ± 10.6 (mean ± standard deviation, the same below) years (*p*=0.010), which is the time from onset to diagnosis. In addition, the duration and times of follow-up were 4.9 (1.8, 7.1) (median, quantile, the same below) years and 4 (2, 6) times, varying from 0.5–9.8 years and 1–10 times, respectively. 28 of their serum Cp concentrations were ≥200 mg/L, and the distribution in each group was not different (*p*=0.784).

The K–F ring positive rate was the highest in HN-type patients (100%), followed by N-type (87.5%) and H-type (77.9%) patients (*p*=0.001). Similarly, liver metabolic indicators of the H-type group, such as ALT, AST, *γ*-glutamyltransferase (*γ*-GT), and prothrombin time (PT), were significantly higher than those of the HN-type and N-type groups (*p* < 0.001, *p*=0.001, 0.025, 0.021). Overall, 57 (66.3%) patients were complicated with LC, of whom 6 (7.0%) patients died due to complications of LC during follow-up, including liver failure, upper gastrointestinal bleeding, hepatic encephalopathy, and infection. Most of the other biochemical results in the three groups had no significant differences, for example, serum Cp, Sco and Cu, urinary copper, white blood cell and platelet (PLT), TB, and creatinine (Cr) (Table 1).

### 3.2. Mutation Analysis of the *ATP7B* Gene

There were 64 patients (H-type: 43, HN-type: 16, and N-type: 5) with *ATP7B* gene-sequencing results. We found 115 mutations (107 missense, 5 frameshift, and 3 splicing site) of 47 sites in 16 exons (Figure S1), which had 6 homozygous, 43 compound heterozygous, 13 heterozygous, and 2 negative. Four patients were found to have three variants. The mutation hotspots were p.R778L (exon 8), P992L (exon 13) and p.R919G (exon 12) (Figure S1, Table S1). The mutation hotspots of the patients, whose serum Cp concentrations were always higher than 140 mg/L (H-type: 22, HN-type: 5, and N-type: 1, data not shown) or fluctuated approximately 140 mg/L (H-type: 19, HN-type: 11, and N-type: 4, data not shown), were not significantly different (*p*=0.750 and 0.332) (Table S1).

### 3.3. Factors Associated with Cp Changes in the WD Cohort

The 352 follow-up records of 86 WD patients were grouped according to serum Cp levels (group A1 vs. A2, Figure 1) in order to identify factors associated with CP changes.

The clinical and biochemical findings of groups A1 and A2 are shown in Table 2. During follow-up, serum Cp fluctuated in most patients according to the boundary value ≥140 mg/L: from high to low and back or not (*n* = 22 and 23, data not shown), always at a high level (*n* = 32), and only once recorded (*n* = 12). Linear regression analysis showed that serum copper, Sco, and Cp in 352 medical records had a significant linear correlation (*R*^2^ = 0.769 and 0.775) (Figure S2). The 24-hour urinary copper levels, which were basic and treated with DMPS, were significantly higher in group A1 ((both *p* ≤ 0.001), 137.2 (95.8, 213.1), and 1534.7 (1054.1, 2265.3)) than in group A2 (112.5 (82.7, 147.9) and 983.7 (523.7, 1618.9)). Group A1 significantly differed from group A2 in items such as AST (*p*=0.011), *γ*-GT (*p* ≤ 0.001), albumin (ALB) (*p* ≤ 0.001), and cholesterol (*p*=0.017).

### 3.4. Factors Associated with WD and a Logistic Regression Model

**Figure 2:**
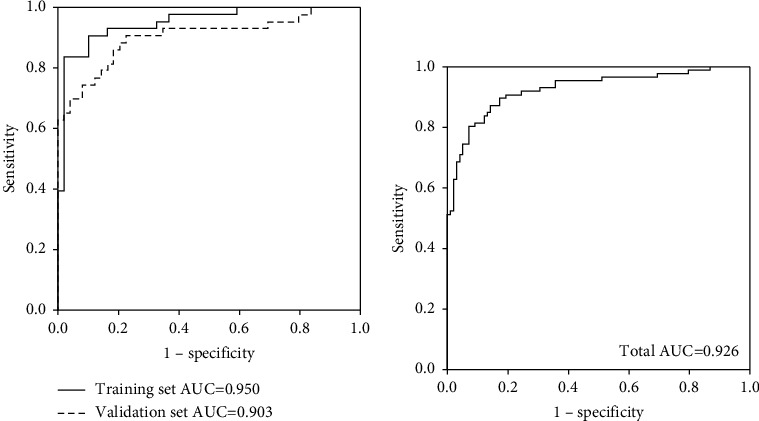
ROC plot of logistic regression model in (a) training and validation set and (b) total set.

For the purpose of differentiating WD patients with nearly normal serum Cp from those with other liver diseases, logistic regression analysis was performed between the training sets of groups A and B. The characteristics of the training and validation sets of groups A and B are listed in Tables 3 and S2, respectively. In the training set, the serum Cp level of group A was significantly lower than that of the control group (group B, *p*=0.002). Most of the liver metabolic indexes of the two groups had no significant difference, according to each factor of univariate binary logistic regression analysis, such as ALT, AST, *γ*-GT, cholinesterase (CHE), ALB, TB, PT, INR, Child–Pugh scores, and proportion of LC, or Child–Pugh classification B and C (*p*=0.075–0.748). Age (*p* ≤ 0.001), AP (*p*=0.040), serum Cr (*p* ≤ 0.001), and cholesterol (Cho, *p*=0.001) revealed a major difference between the two groups. Multivariate binary logistic regression analysis identified 2 variables as independent predictors of WD: age and serum Cr (Table 4), with AUCs of 0.950 (95% CI: 0.907–0.993; *p* ≤ 0.001) in the training set and 0.903 (95% CI: 0.837–0.970; *p* ≤ 0.001) in the validation set (total set: 0.926, 95% CI: 0.887–0.965, and *p* ≤ 0.001) (Figure 2).

The values of this regression model ranged from −13.51 to 11.26. The cutoff value was 0.617, with a sensitivity of 80.2%, specificity of 92.9%, PPV of 90.8%, and NPV of 84.3% in the total cohort to identify WD patients (Table 5). If we use a cutoff value of −1, in the total cohort, the sensitivity of diagnosing WD patients will rise from 80.2% to 90.7%, although its specificity drops 12.3 points to 80.6%. Taking our purpose of reducing missed WD diagnoses into consideration, we thought that it was a reasonable and user-friendly number.

## 4. Discussion

We conducted a retrospective cohort study with the longest follow-up period (9.8 years). The longitudinal study showed that the increased Cp in WD patients is related to the deterioration of liver function indexes and the rise of urinary copper excretion (Table 2). Then, we conducted a case-control study and built a logistic regression model using two easily available markers: age and serum Cr to identify serum Cp-normalised WD from other hepatopathy patients.

This study found that, in WD patients, an increase in the serum Cp concentration mainly occurred when they presented liver function injury or were complicated with LC, such as abnormal changes in AST, *γ*-GT, ALB, cholesterol, and internal copper load (Tables 1 and 2). This is consistent with previous research, in which the serum Cp concentration may be normal in hepatic WD with active liver diseases 12–14 and could decrease to low values after copper chelator treatment 15. By analysing the liver histology of 27 WD patients whose serum Cp levels varied from 210 to 269 mg/L, Peter Ferenci thought that it correlated with liver histologic activity (*r* = 0.47, *p* < 0.05) 15. We found a similar Cp change trend after treatment. At the same time, in our follow-up study, the serum Cp rose again after terminating the treatment. This may be explained by the fact that Cp is mainly produced by the liver and is an acute phase response protein to infection and inflammatory agents [7, 8]. Liver injury caused by the copper burden will increase Cp. This is likely why “normal” Cp is more common in WD patients with significant hepatic damage than in N-type WD patients (Tables 1 and 2) [15].

Cp is the main copper-binding glycoprotein in blood plasma, transporting 40–70% of total serum copper [8]. Serum Cp tested via immunological methods includes biologically inactive apo-Cp and biologically active holo-Cp binding with copper [28]. Sco represents the level of biologically active holo-Cp and has a greater diagnostic value than the Cp concentration determined by immunological techniques [26, 28]. Therefore, we can easily understand that, in the longitudinal study, serum Cu and Sco had significant linear correlations with serum Cp (*R*^*2*^ = 0.769 and 0.775, both *p* ≤ 0.001, Figure S2).

The mutation distribution and allele frequency in the *ATP7B* gene from 64 WD patients in our study show that the hotspots are p.R778L (exon 8), p.P992L (exon 13), and p.R919G (exon 12) (Figure S1), which are in accordance with Chinese prevailing hotspot mutations in WD patients [29]. There was no difference in mutation hotspots between the two group patients whose serum Cp was always higher than 140 mg/L or fluctuated (*p*=0.332) (Table S1). These data did not show that the mutations of the *ATP7B* gene were associated with the change in Cp. The same findings have been found in North American, Russian, and Swedish samples, and their most common WD mutation (p.H1069E) had no significant correlation with ceruloplasmin activity [30].

Serum Cp and K–F rings are the most recognised and easily remembered tests for WD diagnosis. Unfortunately, they are always concealed and puzzling in hepatic-type WD patients 4–6. European guidelines and the Leipzig scoring system 6 have provided a perfect diagnostic pathway for WD diagnosis, but they rely on some parameters such as 24 h urinary copper excretion, *ATP7B* gene, or liver biopsy that are not readily available in ordinary clinical centers and are also expensive and time consuming. In our study, if we calculate the Leipzig score without urinary copper, *ATP7B* gene, and liver biopsy, the sensitivity to diagnose WD is only 64.0% (55/86 patients scored 3), which is obviously lower than that of our model, with a cutoff value of 0.617 (80.2%). Based on the usual indicators, we developed a regression model to recognise these WD patients with “normal” serum Cp, which is helpful and handy.

The parameters (age and serum Cr) adopted in this model are different from those used to identify WD from ALF patients in some other studies, such as AST/ALT and AP/TB [13, 16, 17, 31]. Age and serum creatinine are associated with WD for the following possible reasons. (1) Age: The majority of WD patients present between 5 and 35 years, and liver disease may precede neurologic manifestations by as much as 10 years [6]. In addition, the onset age of other causes of liver diseases is generally later, from 30 to 80 years, for instance, hepatitis B, steatosis hepatitis, and autoimmune hepatitis [32, 33]. Hence, age is a crucial parameter for differentiating WD from other liver diseases. (2) Cr: Nearly all (94%) serum Cr is derived from the healthy muscle and is the end product of creatine and creatine phosphate metabolism [34]. Creatine is synthesized primarily in the liver, and it is then transported to other tissues, particularly skeletal and heart muscles, to be phosphorylated. The serum Cr is produced by a spontaneous, non-enzymatic anhydration of creatine in the muscle cells, which is pH and temperature-dependent. In healthy individuals, the kidney is the main route for Cr elimination. Cr has a low molecular weight, and it is not albumin-bound. Therefore, it is freely filtered at the level of the glomerulus [35]. In our study, the WD patients' serum Cr levels are lower than those in patients with other liver diseases. This difference could not be caused by impaired Cr production due to liver function damage, as Table 3 shows no significant difference in liver function between the two groups. In addition, none of the enrolled patients showed signs of kidney damage, so we believe that a decrease in serum Cr was not caused by kidney damage. Our study did not count muscle mass and 24 h urine Cr of the patients, so it is impossible to judge whether a decrease in Cr in WD patients is related to muscle mass [35, 36]. The accumulation of intracellular copper can induce oxidative stress and perturbing cellular function. Some studies showed that creatine may function as an antioxidant capable of reducing oxidative stress in skeletal myofibers, cardiomyocytes, and hepatocytes [37, 38]. We hypothesize that excessive copper deposition in WD patients leads to overloaded oxidative stress and creatine depletion, which manifests as lower serum creatinine than in other liver diseases. However, whether Cr is directly related to oxidative stress remains unclear.

This logistic regression model has a great AUC value (0.926, Figure 2). We do not insist that our model is perfect, and we recommend that it can only be used for preliminary screening, not definitive diagnosis. Therefore, we selected two cutoff values, one (0.617) with very high PPV and specificity and the other (−1) with very high NPV and sensitivity (Table 5). This means that when you address a cutoff value ≥0.617 to diagnose WD, 90.8% will be correct. Unfortunately, 19.8% WD patients will be missed, due to 80.2% of sensitivity. If we want to increase the sensitivity, −1 may be a better choice. It has a sensitivity of 90.7% and an NPV of 90.8%, which shows that 90.8% of the excluded patients are non-WD patients (Table 5). Overall, both 0.617 and −1 used as the cutoff values for diagnosing and excluding WD have great predictive power. Nevertheless, we still need to be careful with a WD index from −1 to 0.617.

Limitations of our study include that this is a single center and retrospective study which may have bias and decreased the power of our results; the enrolled WD patients were relatively fewer due to it being a rare disease, and fewer patients had elevated serum Cp. In addition, because it is a retrospective study, we cannot obtain all patients' *ATP7B* genes. In fact, the clinical phenotypes, K–F ring, and increased copper excretion were sufficient for the diagnosis of WD. Finally, the pathological mechanism of reduced serum Cr in WD patients is still unclear.

In conclusion, WD patients' increased serum Cp concentrations were mostly pseudo and finally returned to a reasonable level, corresponding to their liver improvement after treatment with a copper chelator, and were not significantly related to *ATP7B* gene mutations. A regression model was established with usual indicators, age and serum Cr, which have good performance in identifying suspicious WD patients from undefined patients whose serum Cp ≥ 140 mg/L. We hope that additional studies in patients who come from multiple centers could further verify this model.

## Figures and Tables

**Table 1:** Baseline characteristics of 86 WD patients with different phenotypes tested in the first hospitalization.

Variables	ALL	Hepatic type	HN-type^†^	Neurologic type	*p* value
Patients (*n*)	86	51	27	8	
Cp ≥ 200 mg/L (*n*, %)	28 (32.6%)	18 (35.3%)	8 (29.6%)	2 (25.0%)	0.784
Sex (male, %)	42 (48.8%)	27 (52.9%)	11 (40.7%)	4 (50%)	0.590
Age at presentation	18.8 (10.8, 28.3)	14.4 (8.0, 23.0)	22.3 ± 9.3	22.5 ± 11.4	0.091
Age at diagnosis	25.1 ± 12.9	21.7 ± 13.0	28.6 ± 9.5	35.0 ± 14.7	0.005
Duration of onset (years)^‡^	2.0 (0.5, 9.3)	1.0 (0.4, 6.2)	3.0 (1.0, 11.1)	12.5 ± 10.6	0.010
Duration of follow-up (years)	4.9 (1.8, 7.1)	4.1 (1.5, 6.9)	5.9 (3.6, 7.5)	3.9 ± 2.6	0.242
Range (years)	0–9.8	0–9.8	0–8.2	0–7.0	N
Total records	352	194	126	32	N
Times per patient	4 (2, 6)	3 (2, 5)	5 (2, 7)	4.0 ± 2.3	0.460
K–F ring (+, %)	67 (77.9%)	33 (64.7%)	27 (100%)	7 (87.5%)	0.001
Serum Cu (*μ*mol/L)	8.6 ± 3.6	8.6 ± 3.7	8.8 ± 3.7	8.0 ± 2.2	0.852
Serum Cp (mg/L)	180.9 (158.4, 214.8)	186.1 (163.6, 215.2)	174.2 (162.0, 209.1)	153.3 (146.1, 212.1)	0.200
Sco (IU/L)	0.211 (0.160, 0.322)	0.211 (0.174, 0.345)	0.223 ± 0.104	0.236 ± 0.110	0.524
Basic urinary copper (*μ*g/24 h)	170.7 (113.7, 408.3)	155.0 (108.9–479.8)	176.4 (126.1, 429.7)	214.1 ± 117.6	0.826
Urinary copper with DMPS (*μ*g/24 h)	2094.6 (1335.4, 3673.1)	1850.3 (1267.5, 4012.5)	2234.3 (1678.4, 3626.2)	1916.6 ± 1015.9	0.399
Complete blood count					
NE (×109/L)	2.4 (1.5, 3.2)	2.2 (1.6, 2.9)	2.3 ± 1.1	3.5 ± 1.2	0.059
PLT (×109/L)	134.5 (81.3, 233.8)	140.0 (79.0, 268.0)	117.0 ± 52.7	199.4 ± 88.1	0.056
RDW (fL)	45.3 ± 7.0	45.5 ± 7.9	45.7 ± 5.8	42.8 ± 2.7	0.551
ALT (IU/L)	43.0 (24.0, 76.8)	60.0 (32.0, 135.0)	28.0 (21.0, 45.0)	36.3 ± 29.6	≤0.001
AST (IU/L)	42.5 (23.8, 71.0)	58.0 (29.0, 84.0)	29.0 (20.0, 39.0)	44.5 (18.0, 70.8)	0.001
*γ*-GT (IU/L)	50.5 (27.0, 108.8)	66.5 (29.0, 151.5)	40.0 (27.0, 91.5)	37.0 ± 26.6	0.025
AP (IU/L)	118.0 (82.0, 241.5)	146.0 (87.3, 303.5)	113.8 ± 56.7	88.0 (85.5, 116.8)	0.030
CHE (IU/L)	5088.4 ± 2536.6	5206.0 (2170.0, 8229.0)	5357.5 (3164.3, 5900.5)	7032.5 (4101.0, 7622.5)	0.277
Albumin (g/L)	40.2 (32.1, 43.7)	37.8 ± 8.5	38.7 ± 6.5	41.8 ± 3.4	0.394
Total bilirubin (*μ*mol/L)	17.0 (11.0, 32.0)	22.3 (9.4, 41.3)	17.3 (13.0, 26.3)	14.1 ± 5.6	0.433
Cr (*μ*mol/L)	47.2 ± 18.6	44.3 ± 18.6	46.8 (39.2, 54.1)	57.4 ± 19.6	0.173
CHO (mmol/L)	4.2 ± 1.2	4.1 ± 1.0	3.8 (3.4, 4.4)	4.8 ± 0.7	0.054
PT (s)	13.6 (12.7, 16.7)	14.1 (12.6, 18.9)	13.5 (13.0, 16.4)	12.3 ± 1.0	0.021
INR	1.1 (1.0, 1.4)	1.1 (1.0, 1.5)	1.1 (1.1, 1.4)	1.0 ± 0.1	0.072
Liver cirrhosis (*n*, %)	57 (66.3%)	30 (58.8%)	27 (100%)	0 (0)	≤0.001
Died (*n*, %)	6 (7.0%)	3 (5.9%)	3 (11.1%)	0 (0)	0.495
Child–Pugh scores	5 (5, 9)	5 (5, 9)	5 (5, 7)	5 (5, 5)	0.051
Class A (*n*)	59	31	20	8	0.116
Class B (*n*)	13	8	5	0	
Class C (*n*)	14	12	2	0	

^†^Hepatic and neurologic presentations; ^‡^from the time of onset to the first hospitalization. The normal distribution parameters are shown as a mean ± standard deviation or else a median and quantile (25%, 75%). K–F ring, Kayser–Fleischer ring; Cp, ceruloplasmin; Sco, serum ceruloplasmin oxidase; DMPS, dimercaptopropane sulfonate; WBC, white blood cell; PLT, platelet; RDW, red cell distribution width; ALT, alanine aminotransferase; AST, aspartate aminotransferase; *γ*-GT, *γ*-glutamyltransferase; AP, alkaline phosphatase; CHE, cholinesterase; Cr, creatinine; CHO, cholesterol; PT, prothrombin time; INR, international normalised ratio.

**Table 2:** Clinical and biochemical findings in 352 hospitalization records of 86 WD patients.

Variables	Group A1	Group A2	*p* ^†^
Records (*n*)	231	121	
Patients (*n*)	86	43	
Hepatic type	51	23	
H-N type	27	16	
Neurologic type	8	4	
Age	27.0 (18.0, 39.0)	25.0 (16.0, 36.0)	0.362
Serum Cu (*μ*mol/L)	8.2 (6.2, 10.2)	3.5 ± 1.6	≤0.001
Serum Cp (mg/L)	186.2 (166.8, 218.4)	94.2 ± 26.9	≤0.001
Sco (IU/L)	0.219 (0.173, 0.336)	0.085 (0.058, 0.115)	≤0.001
Basic urinary copper (*μ*g/24 h)	137.2 (95.8, 213.1)	112.5 (82.7, 147.9)	0.014
Urinary copper with DMPS (*μ*g/24 h)	1534.7 (1054.1, 2265.3)	983.7 (523.7, 1618.9)	≤0.001
Complete blood count			
WBC	5.0 (4.1, 5.9)	5.0 ± 1.5	0.272
PLT (×109/L)	169.0 (111.0, 240.0)	186.1 ± 87.1	0.362
RDW (fL)	42.2 (39.8, 46.4)	43.0 (40.5, 45.7)	0.995
ALT (IU/L)	36.0 (22.0, 55.0)	30.0 (19.0, 46.0)	0.189
AST (IU/L)	31.0 (22.0, 48.0)	26.0 (21.0, 32.5)	0.011
*γ*-GT (IU/L)	38.0 (23.5, 73.5)	22.0 (17.0, 43.0)	≤0.001
AP (IU/L)	112.0 (79.0, 198.0)	102.5 (71.8, 184.0)	0.380
CHE (IU/L)	5734.8 ± 2334.1	5880.8 ± 1439.5	0.717
Albumin (g/L)	42.1 (36.5, 45.0)	43.9 (40.7, 47.5)	≤0.001
Total bilirubin (*μ*mol/L)	13.6 (9.4, 23.3)	14.6 (10.5, 19.7)	0.793
Cr (*μ*mol/L)	49.7 (40.3, 63.0)	53.0 (42.4, 61.2)	0.728
CHO (mmol/L)	4.3 ± 1.1	4.1 ± 0.9	0.017
PT (s)	12.7 (11.7, 14.2)	12.3 (11.9, 13.6)	0.323
INR	1.1 (1.0, 1.2)	1.0 (1.0, 1.1)	0.228
Liver cirrhosis	141 (61.0%)	64 (52.9%)	0.133
Child–Pugh scores	5.0 (5.0, 6.0)	5.0 (5.0, 5.0)	0.997
Class A (*n*)	186	115	1.000^‡^
Classes B and C (*n*)	45	6	

^†^Univariate generalized linear mixed-effects model analysis. ^‡^Child–Pugh classes B and C were merged compared to class A due to the limit of the mixed-effects model. The normal distribution parameters are shown as a mean ± standard deviation or else a median and quantile (25%, 75%). H-N, hepatic-neurologic; Cp, ceruloplasmin; Sco, serum ceruloplasmin oxidase; DMPS, dimercaptopropane sulfonate; WBC, white blood cell; PLT, platelet; RDW, red cell distribution width; ALT, alanine aminotransferase; AST, aspartate aminotransferase; *γ*-GT, *γ*-glutamyltransferase; AP, alkaline phosphatase; CHE, cholinesterase; CB, conjugated bilirubin; Cr, creatinine; CHO, cholesterol; TG, triglycerides; PT, prothrombin time; INR, international normalised ratio.

**Table 3:** Baseline characteristics of WD patients in training sets of groups A and B.

Variables	Group A	Group B	*p* ^†^
*N*	43	49	
Sex (male, %)	25 (58.1%)	38 (77.6%)	0.048
Age	25.3 ± 12.8	50.0 (38.6, 53.5)	≤0.001
Serum Cp (mg/L)	180.3 (152.4, 215.2)	245.5 ± 75.1	0.002
Complete blood count			
WBC (×109/L)	4.8 ± 2.0	4.2 ± 2.0	0.089
PLT (×109/L)	177.7 ± 104.2	103.0 (54.0, 189.0)	0.026
ALT (IU/L)	45.0 (22.0, 96.0)	86.0 (20.5, 183.0)	0.210
AST (IU/L)	46.0 (22.0, 70.0)	44.0 (27.5, 101.0)	0.606
*γ*-GT (IU/L)	54.0 (29.0, 99.0)	68.0 (30.0, 167.5)	0.370
AP (IU/L)	112.5 (84.3, 246.0)	92.0 (77.5, 144.0)	0.040
CHE (IU/L)	5372.1 ± 2551.2	4488.0 (3151.5, 6931.5)	0.806
Albumin (g/L)	39.3 ± 8.2	36.3 ± 8.2	0.091
Total bilirubin (*μ*mol/L)	16.4 (10.9, 26.3)	17.3 (12.0, 45.1)	0.075
Cr (*μ*mol/L)	46.7 ± 18.5	71.1 ± 18.2	≤0.001
CHO (mmol/L)	4.3 ± 0.9	3.5 ± 1.2	0.001
PT (s)	13.4 (12.3, 16.6)	13.9 ± 2.3	0.237
INR	1.1 (1.0, 1.4)	1.2 ± 0.2	0.748
Liver cirrhosis	26 (60.5%)	25 (51.0%)	0.364
Child-Pugh scores	5 (5, 8)	6 (5, 7)	0.638
Class A (*n*)	31	27	0.094
Classes B and C (*n*)	12	22	

^†^ *p* values of univariate binary logistic regression analysis, depending on whether it is WD or not. The normal distribution parameters are shown as a mean ± standard deviation or else a median and quantile (25%, 75%). Cp, ceruloplasmin; WBC, white blood cell; PLT, platelet; ALT, alanine aminotransferase; AST, aspartate aminotransferase; *γ*-GT, *γ*-glutamyltransferase; AP, alkaline phosphatase; CHE, cholinesterase; Cr, creatinine; CHO, cholesterol; PT, prothrombin time; INR, international normalised ratio.

**Table 4:** The performance of binary logistic regression analysis.

Exploratory factor	*β*	SE	*t*	OR	Lower 95(%) limit of OR	Upper 95(%) limit of OR	Significance (*p*)
Constant	13.523	3.081	19.270	746553.8			≤0.001
Age (years)	−0.189	0.044	18.368	0.828	0.760	0.903	≤0.001
Creatinine (*μ*mol/L)	−0.109	0.030	13.627	0.897	0.846	0.950	≤0.001

SE, standard error; OR, odds ratio.

**Table 5:** Accuracy of the logistic regression model.

Group (*N*)	Cutoff value	Sensitivity (%)	Specificity (%)	PPV (%)	NPV (%)
Total (184)	≥0.617	80.2	92.9	90.8	84.3
	≥−1	90.7	80.6	80.4	90.8

Training set (92)	≥0.617	83.7	98.0	97.3	87.3
	≥−1	93.0	81.6	81.6	93.0

Validation set (92)	≥0.617	76.7	87.8	84.6	81.1
	≥−1	88.4	79.6	79.2	88.6

PPV, positive predictive value; NPV, negative predictive value.

## Data Availability

The data that support the findings of this study are available from the corresponding author upon reasonable request.

## Abbreviations

ALB: Albumin
ALF: Acute liver failure
ALT: Alanine aminotransferase
AP: Alkaline phosphatase
AST: Aspartate aminotransferase
AUC: Area under the receiver operating characteristic curve
CB: Conjugated bilirubin
Cp: Ceruloplasmin
Cr: Creatinine
DMPS: Dimercaptopropane sulfonate
*γ*-GT: *γ*-gGlutamyltransferase
GLMM: Generalized linear mixed-effects model
HBV: Hepatitis virus B
INR: International normalised ratio
K–F ring: Kayser–Fleischer ring
LC: Liver cirrhosis
NASH: Nonalcoholic steatosis hepatitis
NPV: Negative predictive value
PLT: Platelet
PT: Prothrombin time
PPV: Positive predictive value
Sco: Serum ceruloplasmin oxidase
TB: Total bilirubin
WD: Wilson's disease.

## Additional Points

*What Is Known*. (i) A decrease in serum ceruloplasmin (Cp) is a crucial marker for the diagnosis of Wilson's disease (WD). (ii) A few WD patients have nearly or completely normal serum Cp, which can lead to missed diagnosis. (iii) There are currently no routine and simple laboratory tests that can identify these WD patients with normalised serum Cp. *What Is New*. (i) The normalisation of serum Cp in WD patients is related to their copper burden and liver function indexes, but not *ATP7B* gene mutation. (ii) Age and serum creatinine are independent risk factors for distinguishing WD from other liver diseases. (iii) Using a regression model built with age and serum creatinine in this study to distinguish undefined WD patients with serum Cp levels ≥140 mg/L has a positive predictive value of 89.3%.

## Supplementary Materials

Supplementary Materials Supplementary tables and figures about the *ATP7B* mutations and baseline characteristics of WD patients in the validation set in this study.
