# Supplementary material for: Age and Serum Creatinine Can Differentiate Wilson Disease Patients with Pseudonormal Ceruloplasmin
Source: Int J Clin Pract. 2023 Mar 2;2023:9344891. doi: 10.1155/2023/9344891 (PMC10008117; doi:10.1155/2023/9344891)
Supplement: Supplementary Materials — Supplementary tables and figures about the ATP7B mutations and baseline characteristics of WD patients in the validation set in this study. [file 9344891.f1.docx]

| **TABLE S1** The *ATP7B* gene mutation hotspots in 62 WD patients | | | | | | |
| --- | --- | --- | --- | --- | --- | --- |
| **Mutation hotspots** | **Cp≥140mg/L (n=28†)** | |  | **Cp fluctuate (n=34†)** | | ***P* value** |
|  | **Position** | **n (total=52‡)** |  | **Position** | **n (total=63‡)** |  |
| 1st Exon | 8 | 12 (23.1%) |  | 13 | 18 (28.6%) | 0.750 |
| 2nd Exon | 13 | 11 (21.2%) |  | 8 | 13 (20.6%) |  |
| 3rd Exon | 12 | 8 (15.4%) |  | 12 | 10 (15.9%) |  |
| 1st AA site | 778 | 9 (17.3%) |  | 992 | 11 (17.5%) | 0.332^c^ |
| 2nd AA site | 992 | 5 (9.6%) |  | 778 | 10 (15.9%) |  |
| 3rd AA site | 616 | 5 (9.6%) |  | 943 | 5 (7.9%) |  |
| †, The number of WD patients whose serum Cp concentrations were always higher than 140mg/L or fluctuated around 140mg/L. ‡, The frequency of ATP7B mutations. ^c^ Chi-Square test between amino acid mutation sites of 778 and 992. AA, amino acid. | | | | | | |

| **TABLE S2**  Baseline characteristics of WD patients in validation sets of group A and B. | | | |
| --- | --- | --- | --- |
| **Variables** | **Group A** | **Group B** | ***P* value** |
| N | 43 | 49 |  |
| Sex (male, %) | 17 (39.5%) | 36 (73.5%) | 0.001 |
| Age | 23.0 (15.0, 35.0) | 49.8±12.8 | < 0.001 |
| Serum Cp (mg/L) | 180.4 (163.6, 209.1) | 227.7±63.7 | 0.052 |
| Complete blood count |  |  |  |
| WBC (×10^9^/L) | 4.7±1.7 | 4.2±1.8 | 0.155 |
| PLT (×10^9^/L) | 136.7±79.3 | 122.6±67.9 | 0.402 |
| ALT (IU/L) | 37.0 (25.0, 66.0) | 85.0 (30.0, 223.5) | 0.010 |
| AST (IU/L) | 37.0 (24.0, 71.0) | 54.0 (33.0, 132.5) | 0.020 |
| γ-GT (IU/L) | 43.0 (24.0, 118.0) | 75.0 (49.5, 136.5) | 0.092 |
| AP (IU/L) | 129.0 (79.8, 240.5) | 101.0 (79.5, 148.5) | 0.152 |
| CHE (IU/L) | 5208.5 (2187.3, 6138.5) | 5138.0 (3605.0, 7679.5) | 0.190 |
| Albumin (g/L) | 39.5 (32.0, 42.8) | 36.8±8.3 | 0.508 |
| Total bilirubin (umol/L) | 17.9 (11.3, 35.3) | 20.0 (14.4, 44.5) | 0.234 |
| Cr (umol/L) | 44.5 (35.3, 55.5) | 71.0±22.2 | < 0.001 |
| CHO (mmol/L) | 3.9 (3.2, 4.8) | 3.7±1.2 | 0.394 |
| PT (s) | 13.8 (12.7, 18.0) | 13.9 (12.1, 15.5) | 0.441 |
| INR | 1.1 (1.1, 1.4) | 1.2±0.2 | 0.527 |
| Liver cirrhosis | 31 (72.1%) | 28 (57.1%) | 0.136 |
| Child-Pugh scores | 5 (5, 9) | 6 (5, 8) | 0.706 |
| class A (n) | 28 | 29 | 0.559 |
| class B&C (n) | 15 | 20 |  |
| The normal distribution parameters are shown as mean±standard deviation, or else median and quantile (25%, 75%). | | | |
| Cp, ceruloplasmin. WBC, white blood cell. PLT, platelet. ALT, alanine aminotransferase. AST, aspartate aminotransferase. γ-GT, γ-glutamyltransferase. AP, alkaline phosphatase. CHE, cholinesterase. CB, conjugated bilirubin. Cr, creatinine. PT, prothrombin time. INR, international normalised ratio. | | | |

**
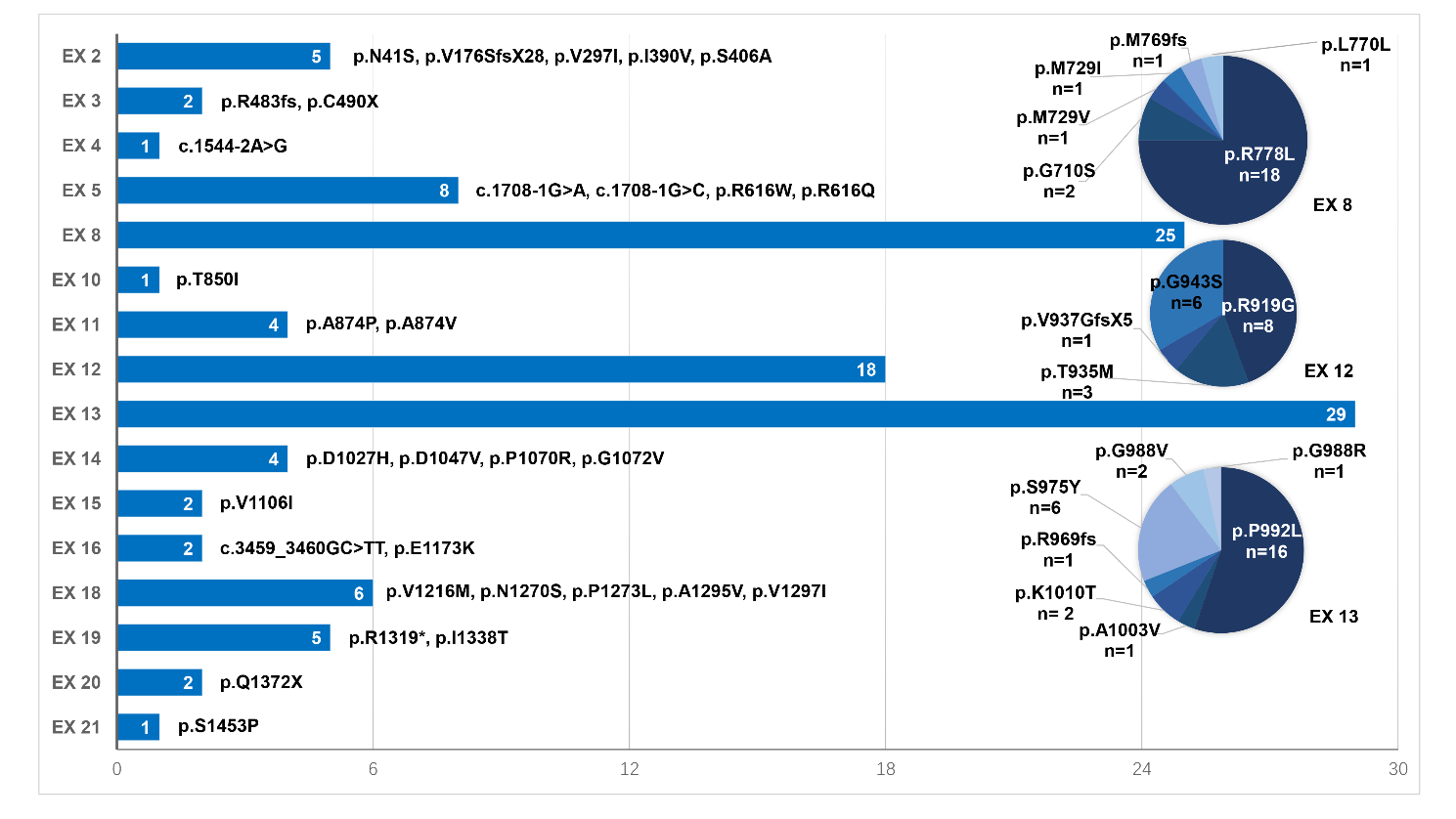
**

**FIGURE S1**  The allele frequencies and distributions of 115 mutations of *ATP7B* gene in 62 WD Patients. The bars show the mutation frequencies of each exon (EX), and the pie charts represent the mutation frequencies, proportions on exons 8, 12 and 13.


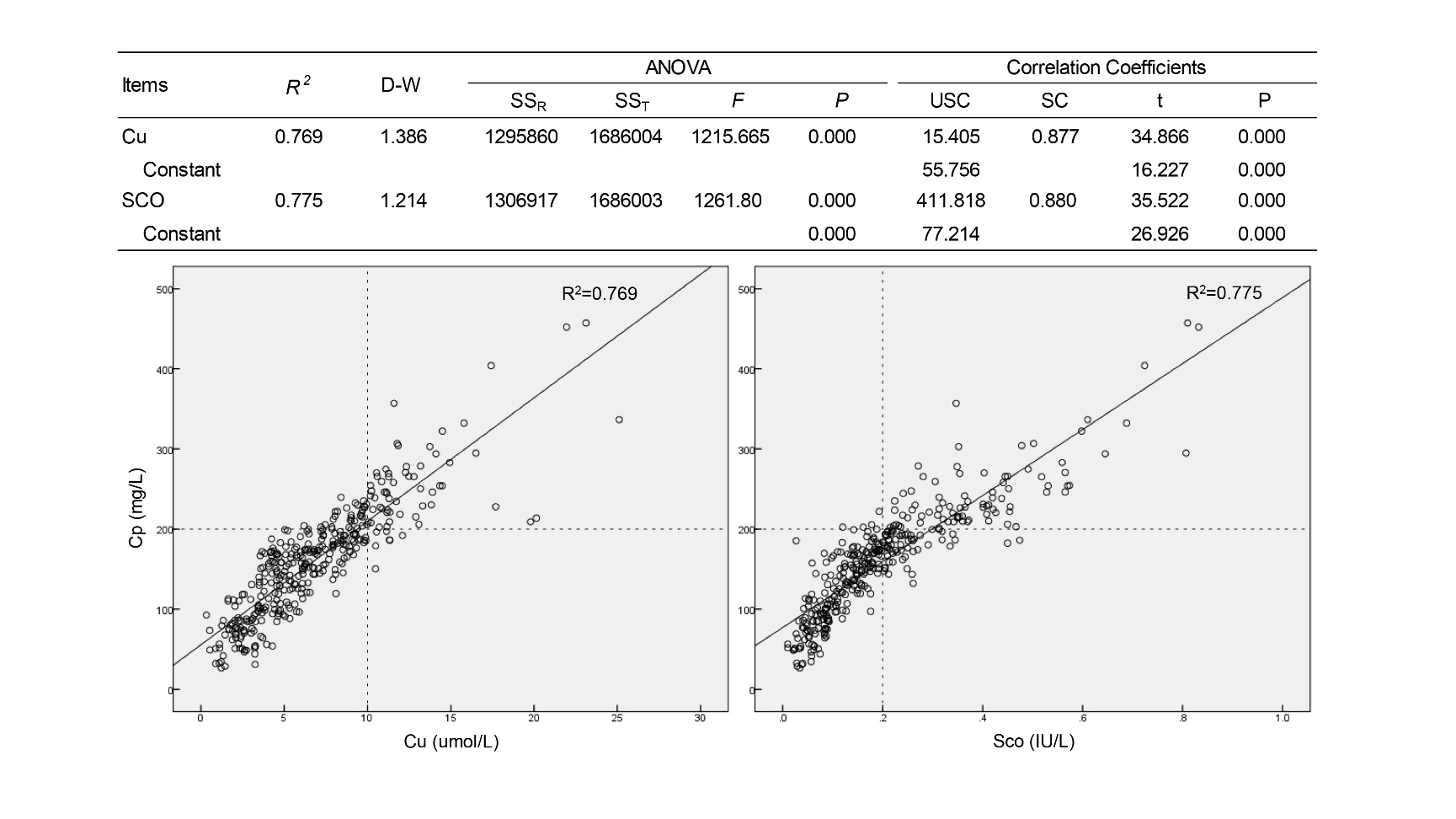


**FIGURE S2** The linear regression performance and scatterplot of serum Cp with serum Cu and Sco respectively. The dotted lines indicate the low limit of normal values of serum Cp (200 mg/L), Cu (10 umol/L) and Sco (0.2 IU/L).
